# Serologic response to porcine circovirus type 1 (PCV1) in infants vaccinated with the human rotavirus vaccine, *Rotarix*™: A retrospective laboratory analysis

**DOI:** 10.1080/21645515.2016.1231262

**Published:** 2016-09-22

**Authors:** Htay Htay Han, Naveen Karkada, Girish Jayadeva, Gary Dubin

**Affiliations:** a GSK Vaccines, King of Prussia, PA, USA; b GlaxoSmithKline Pharmaceuticals Pvt. Ltd., Bangalore, India

**Keywords:** human rotavirus vaccine, *Rotarix*™, porcine circovirus type 1, PCV1, serology

## Abstract

In 2010, porcine circovirus type 1 (PCV1) material was unexpectedly detected in the oral live-attenuated human rotavirus (RV) vaccine, *Rotarix™* (GSK Vaccines, Belgium). An initial study (NCT01511133) found no immunologic response against PCV1 in 40 vaccinated infants. As a follow-up, the current study (NCT02153333), searched for evidence of post-vaccination serologic response to PCV1 in a larger number of archived serum samples. Unlike the previous study, serum anti-PCV1 antibodies were assessed with an adapted Immuno Peroxidase Monolayer Assay (IPMA) using a Vero-adapted PCV1 strain. Samples from 596 infants who participated in clinical trials of the human RV vaccine were randomly selected and analyzed. The observed anti-PCV1 antibody seropositivity rate 1–2 months post-dose 2 was approximately 1% [90% Confidence Interval (CI): 0.3–2.6] (3/299 samples) in infants who received the human RV vaccine and 0.3% [90% CI: 0.0–1.6] (1/297 samples) in those who received placebo; the difference between the groups was −0.66 [90% CI: −2.16–0.60]. One subject in the vaccinated group was also seropositive before vaccination. Notably, the seropositivity rate observed in vaccinated subjects was below that observed during assay qualification in samples from unvaccinated subjects outside of this study (2.5%; 5/200 samples). No serious adverse events had been reported in any of the 4 subjects providing anti-PCV1 positive samples during the 31-day post-vaccination follow-up period in the original studies. In conclusion, the presence of PCV1 in the human RV vaccine is considered to be a manufacturing quality issue and does not appear to pose a safety risk to vaccinated infants.

## Introduction

Rotavirus (RV) is the most common cause of acute gastroenteritis in infants and young children worldwide.^1^ Before the introduction of the currently available RV vaccines, RV gastroenteritis accounted for approximately 2 million hospitalizations and 500,000 deaths annually among children younger than 5 y.^2,3^ Vaccination is considered to be the most effective public health measure to prevent RV infection and reduce the burden of disease and since 2009 the World Health Organization has recommended that RV vaccination should be included in national infant immunization programs.^4^

The oral live-attenuated human RV vaccine (*Rotarix™*, GSK Vaccines, Belgium) has been shown to be efficacious and well-tolerated for preventing severe RV gastroenteritis in large-scale clinical trials undertaken in Latin America, Europe, Asia, Africa, Japan and China.^5-12^ Furthermore, considerable reductions in hospital admissions and mortality due to RV gastroenteritis and all-cause diarrhea have been achieved in various settings around the world following inclusion of the vaccine into national infant immunization schedules.^13-20^ From launch until December 2015, it is estimated that 310 million doses were distributed to markets worldwide.

In January 2010, an independent academic research team utilizing novel and highly sensitive analytical technology unexpectedly identified adventitious agents in some RV vaccines. Porcine circovirus type 1 (PCV1) DNA was detected in the human RV vaccine, *Rotarix*™ (GSK Vaccines, Belgium).^21^ PCV1, a small (<20 nm), non-enveloped DNA virus, containing a single-stranded circular genome,^22^ was first discovered in 1974 as a non-pathogenic contaminant of a porcine kidney cell line.^23-25^ PCV1 infection is asymptomatic in pigs.^26^ Humans are frequently exposed to PCV1 through contaminated meat,^27^ and PCV1 DNA has been detected in human feces and raw sewage.^27,28^ Nevertheless, PCV1 has not been reported to cause infection in humans.^29^

GSK rapidly initiated an investigation to confirm the source, nature and amount of PCV1 in the vaccine manufacturing process and to assess any potential clinical implications.^30^ Results confirmed the presence of PCV1 DNA and low levels of PCV1 viral particles at all stages of the vaccine manufacturing process. It was shown that PCV1 DNA had been present in the vaccine since its initial development, as well as in the vaccine lots used in the pre- and post-licensure clinical trials. When tested in human cell lines, productive PCV1 infection was not observed.^30,31^ Initial retrospective analysis of serum and stool samples from a subset of 40 infants who had received the human RV vaccine in clinical trials revealed no evidence of PCV1 replication and/or immune response to anti-PCV1 antibodies in any of the post-vaccinated infants.^30^ Porcine-derived trypsin, a reagent used during vaccine production in cell culture, appears to be the most likely source of the PCV1 DNA detected in the human RV vaccine.^30^ When the master cell bank was originally generated in 1983, porcine-derived trypsin was not routinely irradiated. Contaminated porcine-derived trypsin was also found to be the source of the low-level contamination of another licensed live-attenuated rotavirus vaccine (*RotaTeq*™, Merck and Co., Inc.) with PCV1 and the closely-related porcine circovirus type 2 (PCV2) DNA fragments.^32^ PCV2 has been linked to post-weaning multi-systemic wasting syndrome and other diseases in pigs;^33-35^ however, there is currently no evidence to suggest PCV1 or PCV2 pathogenicity in humans.^31,36-38^

In order to confirm the initial findings, the US Food and Drug Administration requested GSK to perform a retrospective study on a larger number of archived serum samples with an improved sensitive adapted Immuno Peroxidase Monolayer Assay (IPMA), using the PCV1 strain found in the vaccine (PCV1 strain cultured in Vero cells). The aim of the investigation (NCT02153333) was to detect anti-PCV1 antibodies induced by the PCV1 present in the vaccine. The potential immune response to PCV1 was retrospectively assessed in archived serum samples taken from infants who had received either human RV vaccine or placebo during 6 clinical trials of the human RV vaccine.

## Results

### Study population

**Figure 1.**
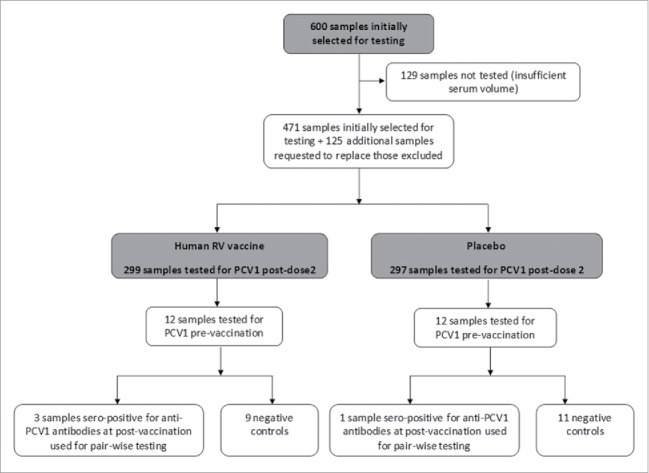
Number of samples selected and tested.

The number of samples randomly selected and tested in this study is summarized in Fig. 1. Of the 600 subjects initially selected to provide samples (with balanced 1:1 randomization from the participating studies), 129 had insufficient serum for testing, therefore an additional 125 subjects were randomly identified for inclusion in this analysis. As replacements were not feasible for 4 subjects (human RV vaccine group: 1; placebo: 3) due to non-availability of subjects with enough serum samples in the respective studies, samples from 596 subjects were finally included (299 in the human RV vaccine group and 297 in the placebo group Table S1. The mean (SD) age of the subjects (randomly selected for testing) at the time of first vaccination was 9.7 (2.4) weeks (Table 1); 50.5% were male, 59.1% were Caucasian, 23.2% were Hispanic and 8.1% were Asian.

**Table 1.** Demographics of the subjects included in analysis.

	Pre-vaccination			Post-vaccination		
Number of subjects	HRV group	Placebo group	Total	HRV group	Placebo group	Total
Planned, N	300	300	600	300	300	600
N	12	12	24	299	297	596
Females:Males	7:5	6:6	13:11	150:149	145:152	295:301
Mean Age, weeks (SD)^*^	10.1 (2.6)	10.0 (2.1)	10.0 (2.3)	9.8 (2.4)	9.5 (2.3)	9.7 (2.4)
Median Age, weeks^*^ (minimum, maximum)	11 (6, 13)	11 (6, 12)	11 (6,13)	10 (6, 14)	9 (6, 16)	9 (6, 16)
White – Caucasian / European Heritage, n (%)	7 (58.3)	6 (50.0)	13 (54.2)	177 (59.2)	175 (58.9)	352 (59.1)
Hispanic, n (%)	2 (16.7)	3 (25.0)	5 (20.8)	70 (23.4)	69 (23.2)	139 (23.2)
Asian – East Asian Heritage, n (%)	0 (0)	1 (8.3)	1 (4.2)	25 (8.4)	23 (7.7)	48 (8.1)

HRV: Human Rotavirus Vaccine group; Placebo: Placebo group; N: Number of subjects tested for anti-PCV1 antibodies; n (%): number (percentage) of subjects in a given category.

* At first dose

### Immunogenicity analysis

The seropositivity rates for anti-PCV1 antibody before and at 1–2 months after the second dose for each study and overall is shown in Table 2. The difference between the 2 groups (placebo group minus human RV vaccine group) post-dose 2 was −0.66 [90% confidence interval (CI): −2.16–0.60]. The difference in terms of seropositivity rates in initially seronegative subjects between the 2 groups post-dose 2 was −0.33 [90% CI: −1.70–0.89].

**Table 2.** Seropositivity rates for anti-PCV1 antibody in sera collected before vaccination and 1–2 months after the second dose by study and overall.

			Seropositivity rates for anti-PCV1-antibody				
						90% CI	
Study	Group	Timing	N	n	%	LL	UL
Rota-005	HRV	Pre	7	0	0.0	0.0	34.8
		Post	7	0	0.0	0.0	34.8
	Placebo	Pre	4	0	0.0	0.0	52.7
		Post	4	0	0.0	0.0	52.7
Rota-023	HRV	Pre	105	1	1.0	0.0	4.4
		Post	105	1	1.0	0.0	4.4
	Placebo	Pre	105	0	0.0	0.0	2.8
		Post	105	1	1.0	0.0	4.4
Rota-028	HRV	Pre	1	0	0.0	0.0	95.0
		Post	1	0	0.0	0.0	95.0
	Placebo	Pre	1	0	0.0	0.0	95.0
		Post	1	0	0.0	0.0	95.0
Rota-029	HRV	Pre	24	0	0.0	0.0	11.7
		Post	24	0	0.0	0.0	11.7
	Placebo	Pre	25	0	0.0	0.0	11.3
		Post	25	0	0.0	0.0	11.3
Rota-036	HRV	Pre	121	0	0.0	0.0	2.4
		Post	121	1	0.8	0.0	3.9
	Placebo	Pre	121	0	0.0	0.0	2.4
		Post	121	0	0.0	0.0	2.4
Rota-054	HRV	Pre	41	0	0.0	0.0	7.0
		Post	41	1	2.4	0.1	11.1
	Placebo	Pre	41	0	0.0	0.0	7.0
		Post	41	0	0.0	0.0	7.0
Overall	HRV	Pre	299	1	0.3	0.0	1.6
		Post	299	3	1.0	0.3	2.6
	Placebo	Pre	297	0	0.0	0.0	1.0
		Post	297	1	0.3	0.0	1.6

HRV: Human Rotavirus Vaccine group; Placebo: Placebo group; N: total number of samples; n/%: number / percentage of samples in a given category; 90% CI: 90% confidence interval; LL: lower limit; UL: upper limit.

Four subjects were seropositive for PCV1 post-vaccination (human RV vaccine group: 3 placebo group: 1), of whom one in the human RV vaccine group was also seropositive for PCV1 before vaccination (Table 2). No statistically significant increase in seropositivity rate was observed after human RV vaccination as compared to the placebo arm.

Among the 4 infants who were seropositive at post-vaccination, 3 had anti-PCV-1 antibodies detected in samples diluted up to 1:120, and the last one up to 1:90. The infant who was seropositive at pre-vaccination had anti-PCV-1 antibodies detected in samples diluted up to 1:120.

### Safety analysis

None of the 4 subjects, who provided samples seropositive for anti-PCV1-antibodies after administration of human RV vaccine or placebo, reported a serious adverse event (SAE) from dose 1 up to the post-dose 2 blood sampling time point.

## Discussion

This retrospective, blinded laboratory evaluation study used a Vero-adapted PCV1 virus IPMA assay to analyze samples taken 1 to 2 months after the second dose of either human RV vaccine or placebo. We observed a post-vaccination anti-PCV1 antibody seropositivity rate of 1% [90% CI: 0.3–2.6] in recipients of the human RV vaccine (3/299 samples) and 0.3% [90% CI: 0.0–1.6] in the placebo group (1/296 samples). The difference in post-vaccination seropositivity rates between the 2 study groups was −0.66% [90% CI: −2.16–0.60]; the difference in terms of post-vaccination seropositivity rates in initially seronegative subjects was −0.33 [90% CI: −1.70–0.89]. The 90% CI for the group difference for overall post-vaccination seropositivity and post-vaccination seropositivity in initially seronegative subjects included 0, indicating that there was no statistically significant increase as compared to the placebo. Of the seropositive subjects, one in the human RV vaccine group had been seropositive before vaccine administration. Since 10-week-old infants have a limited exposure to PVC1, the origin of the anti-PCV1 antibodies measured in this infant is unknown. However, a potential explanation for this unexpected observation could be that this child had maternally-transferred antibodies. Alternatively, this finding could reflect lack of assay specificity. Notably, our observed post-vaccination anti-PCV1 antibody seropositivity rates in both groups in this study are well below the 2.5% rate recorded in a non-vaccinated population (5/200 samples) during the Vero-adapted PCV1 IPMA assay qualification process that was outside the scope of this study. Further, the original investigation conducted by GSK following the detection of PCV1 in human RV vaccine, using an adapted IPMA assay developed to detect anti-PCV1 immunoglobulins in swine, found no evidence of antibodies against PCV1 in serum samples from vaccinated infants.^30^ Briefly, the assay used in the initial study was based on the use of porcine kidney cells (PK15) persistently infected with PCV1 (ATCC-CCL33 strain). In contrast, the IPMA assay used in this study contains the Vero-adapted PCV1 strain found in the human RV vaccine. Eight amino acid changes were observed between capsid sequences of the Vero-adapted and CCL33 PCV1 strains. Assay validation demonstrated that the improved Vero-adapted PCV1 assay has more appropriate repeatability, reproducibility and specificity than the PK15-based assay. More importantly, the Vero-adapted PCV1 assay permits reliable testing of human samples at a lower dilution than the original assay (1/30 compared with 1/100 for the original assay) and this most likely explains why the improved IPMA was able to detect the presence of antibodies that were able to bind PCV1.

The detection of antibodies against animal and plant pathogens in human samples is not unusual: antibodies against swine pasivirus, bovine leukemia virus and tobacco mosaic virus have all been detected in human sera using sensitive methods.^39-41^ Although the origin of anti-PCV1 antibody detection in human samples is not known, PCV1 infection is widespread among pigs, and humans are frequently exposed to PCV1 through the dietary consumption of infected meat products.^25,27,42-44^ Indeed, PCV1 (and/or PCV2) DNA has also been detected in human feces and raw sewage.^27,28,30^ In one US study, 69% of bought pork samples and 5% of human stool samples tested were found to contain PCV1 (and/or PCV2) DNA.^27^ To date, PCV1 has not been shown to cause disease in either pigs or humans, including individuals likely to be at high-risk of infection, such as veterinarians in swine practice.^24,25,36,37^ PCV1 seems unable to productively infect human cell lines.^30,31^ A study from 2011 did suggest productive infection with PCV1 in a sub-clone of human hepatocellular carcinoma cells. The PCV1 replication achieved 10-fold (1 log_10_) lower infectious titer in human cells when compared to the titer normally achieved in porcine kidney cells PK-15.^45^

The presence of adventitious agents in vaccines and/or in the vaccine manufacturing process has been previously reported when animal or plant-derived raw materials are used for production.^21,46,47^ In addition to the presence of porcine circovirus in rotavirus vaccines, there have been reports of nodovirus in the insect cell line used to derive a human papilloma virus vaccine,^47^ avian reovirus in an avian viral vaccine,^48^ pestivirus in a human live viral vaccine,^49^ simian virus 40 (SV40) in Sabin poliovirus vaccine,^50^ and avian leukosis virus and endogenous avian virus in attenuated vaccines grown in chicken embryo fibroblasts.^51,52^

Since retrospective testing has confirmed that PCV1 has been present in the human RV vaccine since the initial stages of its development, the safety data gathered from pre-licensure clinical trials therefore reflect exposure to PCV1 DNA containing vaccine.^30^ Results of an integrated clinical analysis of safety and reactogenicity data summary from 28 randomized, double-blind, placebo-controlled trials involving over 100,000 infants, confirm that the human RV vaccine has a similar safety and tolerability profile to placebo.^53^ Furthermore, none of the subjects seropositive for anti-PCV1 antibodies recorded SAEs during the 31-day follow-up period after vaccination in the original studies. More than 310 million doses of the human RV vaccine have now been distributed worldwide, and no safety risk attributable to the presence of PCV1 DNA in the vaccine has been suggested from extensive post-marketing surveillance.^54-56^

In conclusion, the results of this study using a new PCV1 assay, based on the Vero-adapted PCV1 strain found in *Rotarix™* vaccine, do not demonstrate a significant increase in anti-PCV1 antibody seropositivity rate in infants receiving the human RV vaccine as compared to the placebo recipients. PCV1 is not known to cause disease in either animals or humans and there is no evidence that the presence of PCV1 in the human RV vaccine poses a safety risk. The presence of PCV1 in the human RV vaccine is therefore a manufacturing quality issue.

## Materials and methods

### Study population

Blinded, retrospective laboratory evaluation of archived serum samples, taken from infants who received the human RV vaccine, was undertaken to assess serologic responses to PCV1. The archived serum samples came from subjects who had participated in 6 double-blind, randomized, placebo-controlled trials: ROTA-005 (NCT00729001), ROTA-023 (NCT00140673), ROTA-028 (NCT00197210), ROTA-029 (NCT00197210), ROTA-036 (NCT00140686), and ROTA-054 (NCT00420745).^5,6,57-59^ The studies, conducted in North America, Latin America, Europe and Asia, included healthy infants and were administered the vaccine according to the registered 2-dose schedule (Table 3).

**Table 3.** Overview of clinical studies included in this retrospective analysis.

Study ID (NCT number)	Phase	Location	Population (Schedule)	Blood sample collection time points	Study groups	Reference
ROTA-005 (NCT00729001)	II	USA, Canada	Healthy infants (2, 4 months)	Pre-vaccination and 2 months post-dose 2	Lyophilized HRV 10^6.4^ CCID_50_ Lyophilized HRV 10^5.2^ CCID_50_ Placebo	Dennehy et al. 2005
ROTA-023 (NCT00140673)	III	Argentina, Brazil, Chile, Columbia, Dominican Republic, Honduras, Mexico, Nicaragua, Panama, Peru, Venezuela, Finland	Healthy infants (2, 3–4 months)	Pre-vaccination and 1–2 months post-dose 2	Lyophilized HRV 10^6.5^ CCID_50_ Placebo	Ruiz-Palacios et al. 2006
ROTA-028 (NCT00197210)	III	Singapore	Healthy infants (2, 3–4 months)	Pre-vaccination and 1–2 months post-dose 2	Lyophilized HRV 10^6.5^ CCID_50_ Placebo	Phua et al. 2009
ROTA-029 (NCT00197210)	III	Hong Kong	Healthy infants (2, 3–4 months)	Pre-vaccination and 1–2 months post-dose 2	Lyophilized HRV 10^6.5^ CCID_50_ Placebo	Phua et al. 2009
ROTA-036 (NCT00140686)	IIIb	Czech Republic, Finland, France, Germany, Italy, Spain	Healthy infants (2–3, 3–5 months)	Pre-vaccination and 2 months post-dose 2	Lyophilized HRV 10^6.5^ CCID_50_ Placebo	Vesikari et al. 2007
ROTA-054 (NCT00420745)	IIIb	France, Portugal, Spain, Poland	Pre-term infants (6, 10/14 weeks)	Pre-vaccination and 1–2 months post-dose 2	Lyophilized HRV ≥10^6.0^ CCID_50_ Placebo	Omenaca et al. 2012

HRV: Human Rotavirus Vaccine; CCID_50_: median cell culture infective dose (quantity of virus causing infection in 50% of exposed cells)

Blood samples were collected before vaccination and at 1–2 months post-dose 2. Only serum samples collected from subjects who received 2 doses of either vaccine or placebo and were included in the according-to-protocol cohort for immunogenicity in the original study were eligible for inclusion in this analysis. Initial randomization was performed in each study using an Internet-based central randomization system or a standard Statistical Analysis System (SAS®) program. Samples from 100 subjects (human RV vaccine: 50; placebo: 50) were randomly selected from each of the primary studies for inclusion in this study. If the number of subjects with adequate serum samples was insufficient to meet the target sample size, additional subjects from another study were included to ensure balanced 1:1 randomization.

All primary studies were conducted in accordance with all applicable regulatory requirements and the current testing of anti-PCV1 antibodies was in line with the consent given at the time of the primary studies.

### PCV1 serological assay

All laboratory assays, conducted at GSK Biologicals Clinical Laboratory Sciences, Belgium, were undertaken in a blinded manner, with the individuals responsible for testing being unaware of the study group assignments. The anti-PCV1 antibody response was first assessed in post-vaccination serum samples. If a post-vaccination sample tested negative for anti-PCV1 Immunoglobulin G (IgG) antibodies, then the respective pre-vaccination serum sample from that subject was also assumed to be negative. If a post-vaccination serum sample tested positive for anti-PCV1 antibodies, then paired pre- and post-vaccination serum samples from that subject were tested in an additional run; 20 pairs of pre- and post-vaccination serum samples with negative post-vaccination results, were randomly selected and tested in this additional run as negative controls.

The anti-PCV1 antibody response was assessed using a qualitative Vero-adapted IPMA. Susceptible Vero cells were infected with the Vero-adapted PCV1 strain (PCV1 strain found in the vaccine) and incubated in a 175 cm^2^ T-flask for 3 d at 37°C. The infected cells were seeded in 96-well plates (13,000 cells/well) for an additional 3 d at 37°C, and then fixed and permeabilized using an 80% acetone solution (Merck). Non-infected cell plates were used in parallel as a control for non-specific reactions (PCV1-negative wells). Anti-PCV1-positive monkey control serum and test samples were diluted (1:30, 1:60, 1:90 and 1:120) in an ELISA blocking solution (0.5% Casein blocker from Pierce #37528 + 0.5% BSA solution from KPL #50–61-00) and added to both the PCV1-negative and PCV1-infected cells plates. In addition, the 1:30 and 1:60 serum sample dilutions were spiked with PCV1-positive monkey control serum as an interference control in the most concentrated serum matrix. PCV1-specific antibodies (Immunoglobulins G [IgG]) in the serum bound to any infected cells and were detected after 1 hour incubation at 37°C by adding goat anti-human polyclonal antibodies conjugated to horseradish peroxidase (HRPO, KPL # 214–1002). The HRPO activity was visualized by adding precipitating tetramethylbenzidine peroxidase substrate (True blue, KPL # 50-78-02), which resulted in blue staining of the PCV1-infected cells. When compared to the original PK-15-based assay, the Vero-adapted PCV-1 assay had a lower background (at sample dilution below 1:100) and a higher level of sensitivity.

Seropositivity was defined as anti-PCV1 antibodies detected in any sample diluted ≥ 1:30. The final interpretation (positive or negative) was performed across the 4 serum dilutions and if any sample tested positive, then the subject was considered seropositive for anti-PCV1.

### Statistical analysis

The primary endpoints were the seropositivity rates overall and in initially seronegative subjects for anti-PCV1 antibodies at 1–2 months after the second dose of either the human RV vaccine or placebo. The percentage of PCV1 seropositive subjects post-vaccination and their 2-sided exact 90% CI was tabulated by group for each study and overall (all studies combined).^60^ The difference between the 2 groups with respect to seropositivity rates post-vaccination was calculated with 90% CI.^61^ The analysis was repeated excluding subjects who tested seropositive for anti-PCV1 antibodies before vaccination, to measure the seropositivity rates in initially seronegative subjects.

With a target sample size of 600 serum samples (human RV vaccine: 300; placebo: 300), if all samples tested negative for anti-PCV1 antibody post-dose 2, then the post-vaccination seropositivity rates overall and in initially seronegative subjects would be identical in the 2 groups making it possible to rule out the hypothesis that the difference between the 2 groups in these rates was above 0.9% with 95% confidence (0.9% = upper limit of one-sided 95% CI = upper limit of 2-sided 90% CI). Caution should be taken in the interpretation of this result as no adjustment for multiplicity was done.

## Trademark statement

*Rotarix* is a trademark of the GSK group of companies. *RotaTeq* is a trademark of Merck and Co., Inc.

## Supplementary Material

Supplementary files

## Abbreviations

CI: confidence interval
HRPO: horseradish peroxidase
IPMA: Immuno Peroxidase Monolayer Assay
PCV1: porcine circovirus type 1
RV: rotavirus
SAEs: serious adverse events
