# Supplementary material for: Serologic response to porcine circovirus type 1 (PCV1) in infants vaccinated with the human rotavirus vaccine, Rotarix™: A retrospective laboratory analysis
Source: Hum Vaccin Immunother. 2016 Sep 22;13(1):237–44. doi: 10.1080/21645515.2016.1231262 (PMC5287324; doi:10.1080/21645515.2016.1231262)
Supplement: Supplementary files [file khvi-13-01-1231262-s001.docx]

**Supplementary Table 1.** Number of subjects randomly selected for testing included in this analysis by study

|  | **HRV**  **N = 299** | | **Placebo**  **N = 297** | | **Total**  **N = 596** | |
| --- | --- | --- | --- | --- | --- | --- |
| **Categories** | **n** | **%** | **n** | **%** | **n** | **%** |
|  |  |  |  |  |  |  |
| Rota-005 | 7 | 2.3 | 4 | 1.3 | 11 | 1.8 |
| Rota-023 | 105 | 35.1 | 105 | 35.1 | 210 | 35.2 |
| Rota-028 | 1 | 0.3 | 1 | 0.3 | 2 | 0.3 |
| Rota-029 | 24 | 8.0 | 25 | 8.4 | 49 | 8.2 |
| Rota-036 | 121 | 40.5 | 121 | 40.5 | 242 | 40.6 |
| Rota-054 | 41 | 13.7 | 41 | 13.7 | 82 | 13.8 |
|  |  |  |  |  |  |  |

HRV: Human Rotavirus Vaccine group; Placebo: Placebo group; N: total number of subjects randomly selected for testing; n/%: number / percentage of samples in a given category.
